# Giant Cell Arteritis Presenting as Scalp Necrosis

**DOI:** 10.1100/tsw.2011.123

**Published:** 2011-07-07

**Authors:** Daniel E. Maidana, Silvia Muñoz, Xènia Acebes, Roger Llatjós, Anna Jucglà, Alba Álvarez

**Affiliations:** ^1^ Department of Ophthalmology, Hospital Universitari de Bellvitge, L'Hospitalet de Llobregat, Barcelona, Spain; ^2^ Department of Pathology, Hospital Universitari de Bellvitge, L'Hospitalet de Llobregat, Barcelona, Spain; ^3^ Department of Dermatology, Hospital Universitari de Bellvitge, L'Hospitalet de Llobregat, Barcelona, Spain

**Keywords:** giant cell arteritis, scalp necrosis, temporal artery biopsy

## Abstract

The differential of scalp ulceration in older patients should include several causes, such as herpes zoster, irritant contact dermatitis, ulcerated skin tumors, postirradiation ulcers, microbial infections, pyoderma gangrenosum, and giant cell arteritis. Scalp necrosis associated with giant cell arteritis was first described in the 1940s. The presence of this dermatological sign within giant cell arteritis represents a severity marker of this disease, with a higher mean age at diagnosis, an elevated risk of vision loss and tongue gangrene, as well as overall higher mortality rates, in comparison to patients not presenting this manifestation. Even though scalp necrosis due to giant cell arteritis is exceptional, a high level of suspicion must be held for this clinical finding, in order to initiate prompt and proper treatment and avoid blindness.

Giant cell arteritis is a large and medium-vessel systemic vasculitis that affects predominantly the internal and external carotid arteries, holding a particular tropism for their extracranial branches, especially the superficial temporal artery. The inflammatory process affecting these vessels starts in the adventitia and as it progresses, may lead to vascular lumen narrowing or complete occlusion secondary to proliferation of the intima, and thrombosis. Therefore, ischemic complications develop in tissues irrigated by these vessels, sorting from scalp hypersensitivity to skin necrosis, the latter resulting from the occlusion of the majority of the four arteries irrigating the temporal region of the scalp[1].

A 70-year-old man was referred to the Dermatology outpatient clinic for suspected *tinea capitis*. Past medical history included systemic hypertension, chronic obstructive pulmonary disease, and peripheral vascular disease (Fontaine Stage 1)

He presented with a painful, 6- × 6-cm, necrotic ulceration in the left temporoparietal region of the scalp (Fig. 1.1). There was a smaller-sized satellite lesion with identical characteristics in the occipital area. Both lesions had rapidly developed over the past 6 weeks. He further complained of jaw claudication; however, no fever, malaise, headache, or visual loss was reported.

Blood tests disclosed mild thrombocytosis, ESR was 30 mm/h (range: 2–37 mm). Temporal artery biopsy was performed by the Ophthalmology Department. Histological examination was consistent with giant cell arteritis, evidencing multinucleated giant cells at the media-intima border (Fig. 2). Treatment with oral prednisone (1 mg/kg/day) produced a rapid improvement of his symptoms and a gradual resolution of the ulcerated areas. At 3 months follow-up, the lesions had totally resolved, leaving an alopecic area (Fig. 1.2).

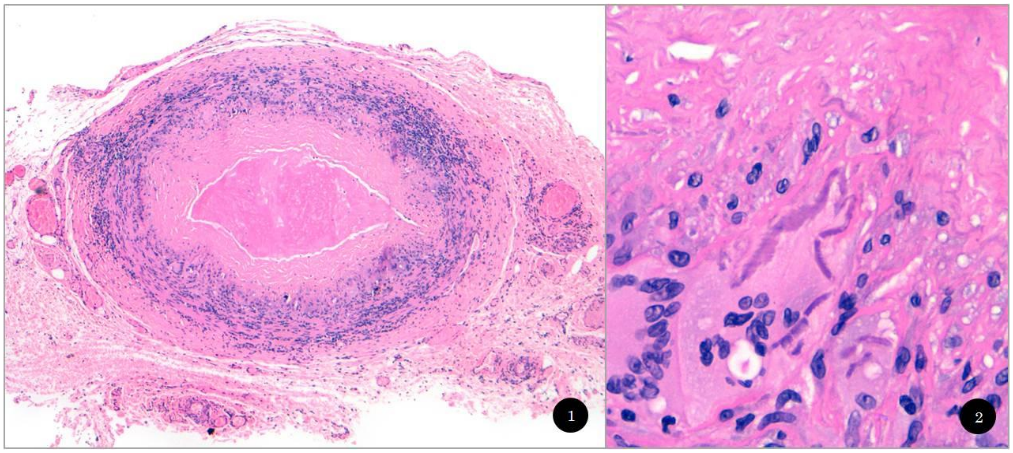

**FIGURE 2.** (1) Temporal artery cross-section (H&E; magnification ×40). (2) Giant cell engulfing fragmented elastic fibers (H&E; magnification ×400).

The differential diagnosis of scalp ulceration in older patients should include several causes, such as herpes zoster, irritant contact dermatitis, ulcerated skin tumors, postirradiation ulcers, microbial infections, pyoderma gangrenosum, and giant cell arteritis[2].

Scalp necrosis associated with giant cell arteritis was first described in the 1940s[3]. The presence of this dermatological sign within giant cell arteritis represents a severity marker of this disease, with a higher mean age at diagnosis[1], an elevated risk of vision loss and tongue gangrene[4], as well as overall higher mortality rates[5], in comparison to patients not presenting this manifestation. Even though scalp necrosis due to giant cell arteritis is exceptional, a high level of suspicion must be held for this clinical finding in order to initiate prompt and proper treatment and avoid blindness.

## References

1. Tsianakas, A., Ehrchen, J.M., Presser, D., Fischer, T., Kruse-Loesler, B., Luger, T.A., and Sunderkoetter, C. (2009) Scalp necrosis in giant cell arteritis: case report and review of the relevance of this cutaneous sign of large-vessel vasculitis. *J. Am. Acad. Dermatol*. **61,** 701–706.
2. Gkalpakiotis, S., Arenberger, P., Sach, J., and Arenbergerova, M. (2011) Temporal arteritis with scalp ulceration and blindness. *J. Dtsch. Dermatol. Ges*. **9,** 50–52.
3. Cooke, W.T., Cloake, P.C.P., Govan, A.D.T., and Colbeck, J.C. (1946) Temporal arteritis: a generalised vascular disease. *Q. J. Med*. **15,** 47–75
4. Currey, J. (1997) Scalp necrosis in giant cell arteritis and review of the literature. *Br. J. Rheumatol.* **36,** 814–816.
5. Soderstrom, C.W. and Seehafer, J.R. (1976) Bilateral scalp necrosis in temporal arteritis: a rare complication of Horton's disease. *Am. J. Med*. **61,** 541–546.
